# (2,2′-Bipyridine-κ^2^ *N*,*N*′)bis­(*N*-isopropyl-*N*-methyl­dithio­carbamato-κ^2^ *S*,*S*′)cadmium

**DOI:** 10.1107/S1600536811012414

**Published:** 2011-04-07

**Authors:** Nor Asiken Abdul Wahab, Ibrahim Baba, Mohamed Ibrahim Mohamed Tahir, Edward R. T. Tiekink

**Affiliations:** a School of Chemical Sciences and Food Technology, Faculty of Science and Technology, Universiti Kebangsaan Malaysia, 43600 Bangi, Malaysia; b Department of Chemistry, Universiti Putra Malaysia, 43400 Serdang, Malaysia; c Department of Chemistry, University of Malaya, 50603 Kuala Lumpur, Malaysia

## Abstract

The Cd^II^ atom in the title compound, [Cd(C_5_H_10_NS_2_)_2_(C_10_H_8_N_2_)], exists in an N_2_S_4_ donor set defined by two chelating dithio­carbamate anions as well as a 2,2′-bipyridine ligand. The coordination geometry approximates a trigonal prism. The crystal packing features weak C—H⋯S inter­actions, leading to linear supra­molecular chains along the *a* axis. The primary connections between these are by π–π stacking inter­actions [ring centroid distance between centrosymmetrically related pyridyl rings = 3.7455 (10) Å]. Overall, the crystal structure may be described as comprising double layers of mol­ecules that stack along the *b* axis.

## Related literature

For related structures of pyridyl adducts of cadmium dithio­carbamtes, see: Song & Tiekink (2009 ▶); Broker & Tiekink (2011 ▶); Jamaluddin *et al.* (2011 ▶).
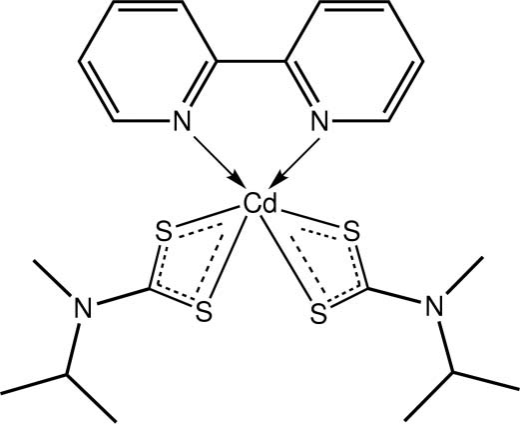


## Experimental

### 

#### Crystal data

[Cd(C_5_H_10_NS_2_)_2_(C_10_H_8_N_2_)]
*M* *_r_* = 565.10
Monoclinic, 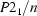

*a* = 9.6061 (2) Å
*b* = 28.6277 (4) Å
*c* = 9.8187 (2) Å
β = 112.860 (2)°
*V* = 2488.07 (8) Å^3^
*Z* = 4
Mo *K*α radiation
μ = 1.23 mm^−1^
*T* = 150 K
0.17 × 0.13 × 0.05 mm

#### Data collection

Oxford Diffraction Xcaliber Eos Gemini diffractometer
Absorption correction: multi-scan (*CrysAlis PRO*; Oxford Diffraction, 2010 ▶) *T*_min_ = 0.853, *T*_max_ = 0.941
53095 measured reflections
5700 independent reflections
5013 reflections with *I* > 2σ(*I*)
*R*_int_ = 0.048

#### Refinement

*R*[*F* ^2^ > 2σ(*F* ^2^)] = 0.022
*wR*(*F* ^2^) = 0.053
*S* = 1.03
5700 reflections
268 parameters
H-atom parameters constrained
Δρ_max_ = 0.42 e Å^−3^
Δρ_min_ = −0.30 e Å^−3^

### 

Data collection: *CrysAlis PRO* (Oxford Diffraction, 2010 ▶); cell refinement: *CrysAlis PRO*; data reduction: *CrysAlis PRO*; program(s) used to solve structure: *SHELXS97* (Sheldrick, 2008 ▶); program(s) used to refine structure: *SHELXL97* (Sheldrick, 2008 ▶); molecular graphics: *ORTEP-3* (Farrugia, 1997 ▶) and *DIAMOND* (Brandenburg, 2006 ▶); software used to prepare material for publication: *publCIF* (Westrip, 2010 ▶).

## Supplementary Material

Crystal structure: contains datablocks global, I. DOI: 10.1107/S1600536811012414/hb5834sup1.cif

Structure factors: contains datablocks I. DOI: 10.1107/S1600536811012414/hb5834Isup2.hkl

Additional supplementary materials:  crystallographic information; 3D view; checkCIF report

## Figures and Tables

**Table 1:** Selected bond lengths (Å)

Cd—S1	2.6463 (5)
Cd—S2	2.7128 (5)
Cd—S3	2.6518 (5)
Cd—S4	2.6490 (5)
Cd—N3	2.4122 (14)
Cd—N4	2.4191 (15)

**Table 2:** Hydrogen-bond geometry (Å, °)

*D*—H⋯*A*	*D*—H	H⋯*A*	*D*⋯*A*	*D*—H⋯*A*
C18—H18⋯S2^i^	0.95	2.78	3.712 (2)	167

Symmetry code: (i) 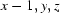.

**Fig. 1.**
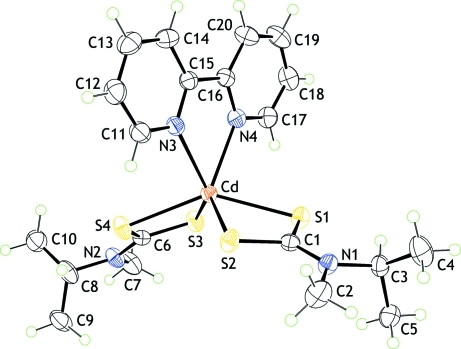
The molecular structure of (I) showing displacement ellipsoids at the 50% probability level.

**Fig. 2.**
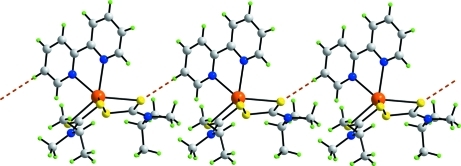
A view of the linear supramolecular chain long the a axis in (I) mediated by C—H···S interactions (orange dashed lines) along the a axis.

**Fig. 3.**
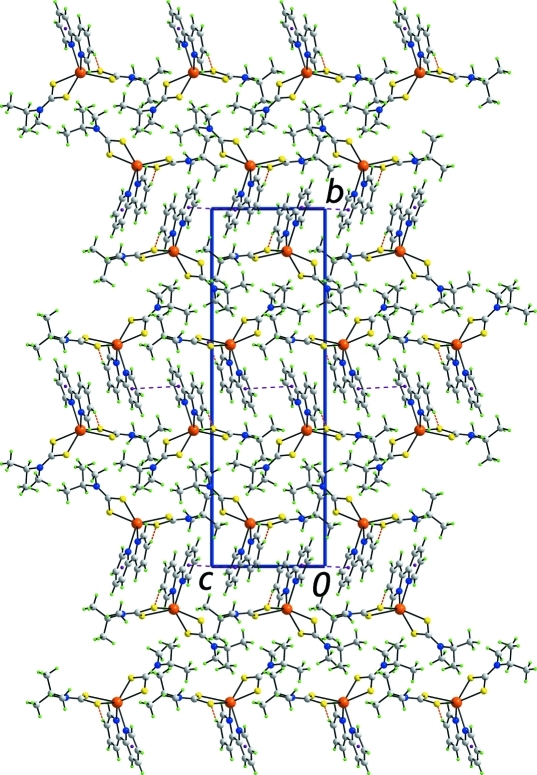
A view in projection down the a axis of the crystal packing in (I) highlighting the π–π interactions (shown as purple dashed lines).

## supplementary crystallographic information

### Comment

In continuation of systematic structural studies of various pyridyl adducts of cadmium dithiocarbamates (Song & Tiekink, 2009; Broker & Tiekink, 2011; Jamaluddin *et al.*, 2011), the title compound Cd[S_2_CN(Me)iPr)_2_]_2_(2,2'-bipyridine), (I), was investigated. The Cd^II^ atom is coordinated by two dithiocarbamate ligands, each essentially forming symmetric Cd—S bonds, and a symmetrically chelating 2,2'-bipyridine ligand, Fig. 1 and Table 1. The equivalence in the Cd—S bond distances is reflected in the narrow range of associated C≐S bond distances, *i.e*. 1.7168 (18) to 1.7290 (17) Å. A small twist is noted between the pyridyl rings of the 2,2'-bipyridine ligand as seen in the dihedral angle of 9.25 (9) ° formed between the rings. The N_2_S_4_ donor set defines a distorted trigonal prismatic geometry.

The crystal packing of (I) features linear supramolecular chains along the *a* axis that are sustained by C—H···S interactions, Fig. 2 and Table 2. Chains lie in the *ac* plane and inter-digitate *via*π–π interactions with centrosymmetrically related layers to form a double layer [ring centroid(N3-pyridyl)···ring centroid(N3-centroid)^i^ = 3.7455 (10) Å for *i*: 2 - *x*, 1 - *y*, 1 - *z*]. Double layers stack along the *b* axis and are separated by hydrophobic interactions, Fig. 3.

### Experimental

The title compound was prepared using an *in situ* method by the addition of carbon disulfide (0.02 mol) to an ethanolic solution (20 ml) of methylisopropropylamine (0.02 mol) and 2,2-bipyridine (0.01 mol) in ethanol (20 ml). The mixture was stirred for 1 h at 277 K. The resulting solution was added drop-wise to a solution of cadmium(II) dichloride (0.01 mol) in ethanol (20 ml). The mixture was stirred 3 h. The yellowish precipitate was filtered, washed with cold ethanol and dried in a desiccator. Recrystallization was from ethanol:chloroform (1:2 *v*/*v*) to yield yellow prisms of (I). *M*.pt. 473.6–475.2 K. Elemental analysis. Found (calculated) for C_22_H_32_CdN_4_S_4_: C, 42.63 (42.51); H 4.48 (4.99); N 10.74 (9.91); S 21.80 (22.70) %. UV (CHCl_3_) λ_max_ 283.5 and 261.0 nm (*L*(π) →*L*(π*)). IR (KBr): ν(C—H) 2928 s; ν(C≐N) 1565 s; ν(N—C) 1468 m; ν(C≐S) 970 s; ν(Cd—S) 381 s cm^-1^.

### Refinement

Carbon-bound H-atoms were placed in calculated positions (C—H 0.95 to 1.00 Å) and were included in the refinement in the riding model approximation, with *U*_iso_(H) set to 1.2 to 1.5*U*_equiv_(C).

### Crystal data

**Table:**

[Cd(C_5_H_10_NS_2_)_2_(C_10_H_8_N_2_)]	*F*(000) = 1152
*M**_r_* = 565.10	*D*_x_ = 1.509 Mg m^−^^3^
Monoclinic, *P*2_1_/*n*	Mo *K*α radiation, λ = 0.71073 Å
Hall symbol: -P 2yn	Cell parameters from 26576 reflections
*a* = 9.6061 (2) Å	θ = 2–29°
*b* = 28.6277 (4) Å	µ = 1.23 mm^−^^1^
*c* = 9.8187 (2) Å	*T* = 150 K
β = 112.860 (2)°	Prism, yellow
*V* = 2488.07 (8) Å^3^	0.17 × 0.13 × 0.05 mm
*Z* = 4	

### Data collection

**Table:**

Oxford Diffraction Xcaliber Eos Gemini diffractometer	5700 independent reflections
Radiation source: fine-focus sealed tube	5013 reflections with *I* > 2σ(*I*)
graphite	*R*_int_ = 0.048
Detector resolution: 16.1952 pixels mm^-1^	θ_max_ = 27.5°, θ_min_ = 2.4°
ω scans	*h* = −12→12
Absorption correction: multi-scan (*CrysAlis PRO*; Oxford Diffraction, 2010)	*k* = −37→37
*T*_min_ = 0.853, *T*_max_ = 0.941	*l* = −12→12
53095 measured reflections	

### Refinement

**Table:**

Refinement on *F*^2^	Primary atom site location: structure-invariant direct methods
Least-squares matrix: full	Secondary atom site location: difference Fourier map
*R*[*F*^2^ > 2σ(*F*^2^)] = 0.022	Hydrogen site location: inferred from neighbouring sites
*wR*(*F*^2^) = 0.053	H-atom parameters constrained
*S* = 1.03	*w* = 1/[σ^2^(*F*_o_^2^) + (0.023*P*)^2^ + 0.9719*P*] where *P* = (*F*_o_^2^ + 2*F*_c_^2^)/3
5700 reflections	(Δ/σ)_max_ = 0.002
268 parameters	Δρ_max_ = 0.42 e Å^−^^3^
0 restraints	Δρ_min_ = −0.30 e Å^−^^3^

### Special details

**Table:**

Geometry. All s.u.'s (except the s.u. in the dihedral angle between two l.s. planes) are estimated using the full covariance matrix. The cell s.u.'s are taken into account individually in the estimation of s.u.'s in distances, angles and torsion angles; correlations between s.u.'s in cell parameters are only used when they are defined by crystal symmetry. An approximate (isotropic) treatment of cell s.u.'s is used for estimating s.u.'s involving l.s. planes.
Refinement. Refinement of *F*^2^ against ALL reflections. The weighted *R*-factor *wR* and goodness of fit *S* are based on *F*^2^, conventional *R*-factors *R* are based on *F*, with *F* set to zero for negative *F*^2^. The threshold expression of *F*^2^ > 2σ(*F*^2^) is used only for calculating *R*-factors(gt) *etc*. and is not relevant to the choice of reflections for refinement. *R*-factors based on *F*^2^ are statistically about twice as large as those based on *F*, and *R*- factors based on ALL data will be even larger.

### Fractional atomic coordinates and isotropic or equivalent isotropic displacement parameters (Å^2^)

**Table:**

	*x*	*y*	*z*	*U*_iso_*/*U*_eq_	
Cd	1.074719 (14)	0.619541 (4)	0.837624 (13)	0.02469 (5)	
S1	1.13178 (5)	0.638504 (17)	1.11803 (5)	0.02979 (10)	
S2	1.37352 (5)	0.615697 (17)	1.01159 (5)	0.03243 (10)	
S3	0.88132 (5)	0.688040 (16)	0.71256 (5)	0.03348 (11)	
S4	1.10292 (5)	0.651618 (17)	0.59657 (5)	0.03368 (11)	
N1	1.42420 (16)	0.63344 (5)	1.29241 (16)	0.0285 (3)	
N2	0.91865 (17)	0.72387 (5)	0.47972 (17)	0.0313 (3)	
N3	1.09345 (17)	0.54076 (5)	0.75929 (16)	0.0282 (3)	
N4	0.87517 (16)	0.56931 (5)	0.84481 (16)	0.0292 (3)	
C1	1.32019 (19)	0.62947 (6)	1.15504 (19)	0.0254 (3)	
C2	1.5853 (2)	0.63217 (9)	1.3206 (2)	0.0448 (5)	
H2A	1.6125	0.6009	1.2987	0.067*	
H2B	1.6444	0.6397	1.4245	0.067*	
H2C	1.6070	0.6551	1.2573	0.067*	
C3	1.3829 (2)	0.64480 (7)	1.41991 (19)	0.0296 (4)	
H3	1.2720	0.6392	1.3881	0.036*	
C4	1.4639 (3)	0.61310 (9)	1.5506 (3)	0.0521 (6)	
H4A	1.4428	0.5804	1.5198	0.078*	
H4B	1.4281	0.6196	1.6296	0.078*	
H4C	1.5729	0.6188	1.5870	0.078*	
C5	1.4115 (2)	0.69603 (7)	1.4595 (2)	0.0414 (5)	
H5A	1.5206	0.7020	1.5016	0.062*	
H5B	1.3688	0.7042	1.5322	0.062*	
H5C	1.3637	0.7151	1.3705	0.062*	
C6	0.96267 (19)	0.69114 (6)	0.58500 (19)	0.0253 (3)	
C7	0.7956 (2)	0.75621 (7)	0.4673 (3)	0.0439 (5)	
H7A	0.7009	0.7388	0.4401	0.066*	
H7B	0.7853	0.7796	0.3912	0.066*	
H7C	0.8185	0.7718	0.5624	0.066*	
C8	0.9824 (2)	0.72780 (7)	0.3644 (2)	0.0374 (5)	
H8	1.0722	0.7067	0.3931	0.045*	
C9	1.0361 (3)	0.77737 (8)	0.3560 (2)	0.0491 (6)	
H9A	0.9485	0.7980	0.3128	0.074*	
H9B	1.0953	0.7777	0.2942	0.074*	
H9C	1.0991	0.7883	0.4557	0.074*	
C10	0.8681 (3)	0.71105 (8)	0.2173 (2)	0.0455 (5)	
H10A	0.8415	0.6785	0.2264	0.068*	
H10B	0.9116	0.7133	0.1423	0.068*	
H10C	0.7772	0.7305	0.1880	0.068*	
C11	1.1989 (2)	0.52925 (7)	0.7083 (2)	0.0359 (4)	
H11	1.2712	0.5522	0.7104	0.043*	
C12	1.2078 (2)	0.48551 (7)	0.6527 (2)	0.0405 (5)	
H12	1.2837	0.4786	0.6162	0.049*	
C13	1.1038 (2)	0.45225 (7)	0.6515 (2)	0.0405 (5)	
H13	1.1069	0.4218	0.6140	0.049*	
C14	0.9948 (2)	0.46353 (6)	0.7053 (2)	0.0349 (4)	
H14	0.9226	0.4409	0.7059	0.042*	
C15	0.99217 (19)	0.50838 (6)	0.75862 (18)	0.0269 (4)	
C16	0.87718 (19)	0.52370 (6)	0.81662 (18)	0.0265 (4)	
C17	0.7754 (2)	0.58528 (7)	0.8983 (2)	0.0355 (4)	
H17	0.7746	0.6177	0.9183	0.043*	
C18	0.6735 (2)	0.55653 (8)	0.9256 (2)	0.0394 (5)	
H18	0.6034	0.5689	0.9627	0.047*	
C19	0.6762 (2)	0.50964 (8)	0.8977 (2)	0.0436 (5)	
H19	0.6081	0.4890	0.9161	0.052*	
C20	0.7787 (2)	0.49272 (7)	0.8427 (2)	0.0387 (4)	
H20	0.7819	0.4603	0.8230	0.046*	

### Atomic displacement parameters (Å^2^)

**Table:**

	*U*^11^	*U*^22^	*U*^33^	*U*^12^	*U*^13^	*U*^23^
Cd	0.02824 (7)	0.02325 (7)	0.02276 (7)	0.00063 (5)	0.01010 (5)	0.00100 (5)
S1	0.0242 (2)	0.0389 (3)	0.0253 (2)	0.00347 (18)	0.00845 (17)	−0.00182 (18)
S2	0.0316 (2)	0.0385 (3)	0.0299 (2)	0.00315 (19)	0.01483 (19)	−0.00517 (19)
S3	0.0408 (3)	0.0309 (2)	0.0352 (3)	0.0088 (2)	0.0218 (2)	0.00636 (19)
S4	0.0373 (2)	0.0349 (2)	0.0347 (2)	0.01276 (19)	0.0204 (2)	0.01318 (19)
N1	0.0226 (7)	0.0347 (8)	0.0274 (8)	−0.0005 (6)	0.0089 (6)	−0.0037 (6)
N2	0.0307 (8)	0.0292 (8)	0.0381 (9)	0.0075 (6)	0.0177 (7)	0.0115 (6)
N3	0.0329 (8)	0.0267 (8)	0.0280 (8)	0.0006 (6)	0.0150 (6)	0.0020 (6)
N4	0.0288 (8)	0.0293 (8)	0.0291 (8)	0.0015 (6)	0.0108 (6)	0.0016 (6)
C1	0.0267 (8)	0.0203 (8)	0.0291 (9)	−0.0001 (6)	0.0109 (7)	0.0008 (6)
C2	0.0228 (9)	0.0629 (14)	0.0460 (12)	0.0012 (9)	0.0103 (9)	−0.0117 (10)
C3	0.0255 (8)	0.0390 (10)	0.0239 (9)	0.0000 (7)	0.0091 (7)	0.0016 (7)
C4	0.0520 (14)	0.0628 (15)	0.0412 (13)	0.0102 (11)	0.0177 (11)	0.0200 (11)
C5	0.0482 (12)	0.0441 (12)	0.0366 (11)	−0.0062 (9)	0.0217 (9)	−0.0101 (9)
C6	0.0257 (8)	0.0213 (8)	0.0284 (9)	−0.0028 (6)	0.0099 (7)	−0.0003 (7)
C7	0.0452 (12)	0.0355 (11)	0.0595 (14)	0.0161 (9)	0.0297 (11)	0.0192 (10)
C8	0.0332 (10)	0.0431 (11)	0.0405 (11)	0.0109 (8)	0.0191 (9)	0.0195 (9)
C9	0.0434 (12)	0.0626 (15)	0.0373 (12)	−0.0150 (11)	0.0115 (10)	0.0146 (10)
C10	0.0553 (13)	0.0397 (12)	0.0475 (13)	0.0006 (10)	0.0265 (11)	0.0006 (9)
C11	0.0420 (11)	0.0308 (10)	0.0415 (11)	0.0001 (8)	0.0234 (9)	0.0012 (8)
C12	0.0468 (12)	0.0376 (11)	0.0445 (12)	0.0083 (9)	0.0259 (10)	0.0009 (9)
C13	0.0465 (12)	0.0287 (10)	0.0446 (12)	0.0072 (9)	0.0160 (10)	−0.0030 (8)
C14	0.0350 (10)	0.0259 (9)	0.0404 (11)	−0.0001 (8)	0.0110 (8)	−0.0009 (8)
C15	0.0291 (9)	0.0249 (9)	0.0231 (8)	0.0017 (7)	0.0061 (7)	0.0044 (7)
C16	0.0248 (8)	0.0267 (9)	0.0248 (9)	0.0025 (7)	0.0062 (7)	0.0052 (7)
C17	0.0343 (10)	0.0365 (10)	0.0370 (11)	0.0046 (8)	0.0154 (8)	0.0001 (8)
C18	0.0298 (10)	0.0513 (13)	0.0397 (11)	0.0089 (9)	0.0164 (8)	0.0089 (9)
C19	0.0337 (10)	0.0461 (12)	0.0551 (13)	0.0011 (9)	0.0216 (10)	0.0161 (10)
C20	0.0355 (10)	0.0303 (10)	0.0513 (12)	0.0015 (8)	0.0181 (9)	0.0084 (9)

### Geometric parameters (Å, °)

**Table:**

Cd—S1	2.6463 (5)	C5—H5C	0.9800
Cd—S2	2.7128 (5)	C7—H7A	0.9800
Cd—S3	2.6518 (5)	C7—H7B	0.9800
Cd—S4	2.6490 (5)	C7—H7C	0.9800
Cd—N3	2.4122 (14)	C8—C10	1.512 (3)
Cd—N4	2.4191 (15)	C8—C9	1.523 (3)
S1—C1	1.7215 (18)	C8—H8	1.0000
S2—C1	1.7212 (18)	C9—H9A	0.9800
S3—C6	1.7168 (18)	C9—H9B	0.9800
S4—C6	1.7290 (17)	C9—H9C	0.9800
N1—C1	1.335 (2)	C10—H10A	0.9800
N1—C2	1.463 (2)	C10—H10B	0.9800
N1—C3	1.488 (2)	C10—H10C	0.9800
N2—C6	1.336 (2)	C11—C12	1.382 (3)
N2—C7	1.469 (2)	C11—H11	0.9500
N2—C8	1.486 (2)	C12—C13	1.377 (3)
N3—C11	1.334 (2)	C12—H12	0.9500
N3—C15	1.342 (2)	C13—C14	1.382 (3)
N4—C16	1.337 (2)	C13—H13	0.9500
N4—C17	1.340 (2)	C14—C15	1.390 (2)
C2—H2A	0.9800	C14—H14	0.9500
C2—H2B	0.9800	C15—C16	1.492 (2)
C2—H2C	0.9800	C16—C20	1.391 (3)
C3—C5	1.515 (3)	C17—C18	1.382 (3)
C3—C4	1.516 (3)	C17—H17	0.9500
C3—H3	1.0000	C18—C19	1.372 (3)
C4—H4A	0.9800	C18—H18	0.9500
C4—H4B	0.9800	C19—C20	1.382 (3)
C4—H4C	0.9800	C19—H19	0.9500
C5—H5A	0.9800	C20—H20	0.9500
C5—H5B	0.9800		
			
N3—Cd—N4	67.15 (5)	N2—C6—S3	120.24 (13)
N3—Cd—S1	120.84 (4)	N2—C6—S4	120.93 (13)
N4—Cd—S1	86.43 (4)	S3—C6—S4	118.83 (10)
N3—Cd—S4	89.60 (4)	N2—C7—H7A	109.5
N4—Cd—S4	126.11 (4)	N2—C7—H7B	109.5
S1—Cd—S4	143.780 (17)	H7A—C7—H7B	109.5
N3—Cd—S3	132.02 (4)	N2—C7—H7C	109.5
N4—Cd—S3	91.86 (4)	H7A—C7—H7C	109.5
S1—Cd—S3	98.905 (15)	H7B—C7—H7C	109.5
S4—Cd—S3	68.057 (14)	N2—C8—C10	110.03 (16)
N3—Cd—S2	88.34 (4)	N2—C8—C9	111.10 (17)
N4—Cd—S2	127.89 (4)	C10—C8—C9	112.31 (16)
S1—Cd—S2	67.192 (14)	N2—C8—H8	107.7
S4—Cd—S2	97.251 (15)	C10—C8—H8	107.7
S3—Cd—S2	134.517 (16)	C9—C8—H8	107.7
C1—S1—Cd	87.98 (6)	C8—C9—H9A	109.5
C1—S2—Cd	85.84 (6)	C8—C9—H9B	109.5
C6—S3—Cd	86.59 (6)	H9A—C9—H9B	109.5
C6—S4—Cd	86.44 (6)	C8—C9—H9C	109.5
C1—N1—C2	120.67 (15)	H9A—C9—H9C	109.5
C1—N1—C3	121.93 (14)	H9B—C9—H9C	109.5
C2—N1—C3	117.02 (14)	C8—C10—H10A	109.5
C6—N2—C7	120.58 (15)	C8—C10—H10B	109.5
C6—N2—C8	122.85 (15)	H10A—C10—H10B	109.5
C7—N2—C8	116.46 (14)	C8—C10—H10C	109.5
C11—N3—C15	118.85 (16)	H10A—C10—H10C	109.5
C11—N3—Cd	120.81 (12)	H10B—C10—H10C	109.5
C15—N3—Cd	120.24 (11)	N3—C11—C12	123.01 (18)
C16—N4—C17	118.99 (16)	N3—C11—H11	118.5
C16—N4—Cd	119.95 (11)	C12—C11—H11	118.5
C17—N4—Cd	120.30 (12)	C13—C12—C11	118.27 (19)
N1—C1—S2	120.15 (13)	C13—C12—H12	120.9
N1—C1—S1	120.87 (13)	C11—C12—H12	120.9
S2—C1—S1	118.97 (10)	C12—C13—C14	119.36 (18)
N1—C2—H2A	109.5	C12—C13—H13	120.3
N1—C2—H2B	109.5	C14—C13—H13	120.3
H2A—C2—H2B	109.5	C13—C14—C15	119.15 (18)
N1—C2—H2C	109.5	C13—C14—H14	120.4
H2A—C2—H2C	109.5	C15—C14—H14	120.4
H2B—C2—H2C	109.5	N3—C15—C14	121.37 (17)
N1—C3—C5	110.28 (15)	N3—C15—C16	115.97 (15)
N1—C3—C4	111.39 (16)	C14—C15—C16	122.66 (16)
C5—C3—C4	112.32 (17)	N4—C16—C20	121.27 (17)
N1—C3—H3	107.5	N4—C16—C15	116.02 (15)
C5—C3—H3	107.5	C20—C16—C15	122.70 (16)
C4—C3—H3	107.5	N4—C17—C18	122.76 (19)
C3—C4—H4A	109.5	N4—C17—H17	118.6
C3—C4—H4B	109.5	C18—C17—H17	118.6
H4A—C4—H4B	109.5	C19—C18—C17	118.32 (19)
C3—C4—H4C	109.5	C19—C18—H18	120.8
H4A—C4—H4C	109.5	C17—C18—H18	120.8
H4B—C4—H4C	109.5	C18—C19—C20	119.48 (19)
C3—C5—H5A	109.5	C18—C19—H19	120.3
C3—C5—H5B	109.5	C20—C19—H19	120.3
H5A—C5—H5B	109.5	C19—C20—C16	119.17 (19)
C3—C5—H5C	109.5	C19—C20—H20	120.4
H5A—C5—H5C	109.5	C16—C20—H20	120.4
H5B—C5—H5C	109.5		
			
N3—Cd—S1—C1	72.53 (7)	Cd—S2—C1—S1	−1.23 (9)
N4—Cd—S1—C1	133.16 (7)	Cd—S1—C1—N1	−179.36 (14)
S4—Cd—S1—C1	−70.74 (6)	Cd—S1—C1—S2	1.26 (9)
S3—Cd—S1—C1	−135.51 (6)	C1—N1—C3—C5	−100.74 (19)
S2—Cd—S1—C1	−0.76 (6)	C2—N1—C3—C5	72.2 (2)
N3—Cd—S2—C1	−123.88 (7)	C1—N1—C3—C4	133.84 (18)
N4—Cd—S2—C1	−64.86 (7)	C2—N1—C3—C4	−53.2 (2)
S1—Cd—S2—C1	0.76 (6)	C7—N2—C6—S3	−2.8 (2)
S4—Cd—S2—C1	146.73 (6)	C8—N2—C6—S3	−178.74 (14)
S3—Cd—S2—C1	80.51 (6)	C7—N2—C6—S4	177.74 (15)
N3—Cd—S3—C6	−69.81 (7)	C8—N2—C6—S4	1.8 (2)
N4—Cd—S3—C6	−130.23 (7)	Cd—S3—C6—N2	−176.65 (14)
S1—Cd—S3—C6	143.09 (6)	Cd—S3—C6—S4	2.79 (9)
S4—Cd—S3—C6	−1.72 (6)	Cd—S4—C6—N2	176.64 (14)
S2—Cd—S3—C6	76.43 (6)	Cd—S4—C6—S3	−2.79 (9)
N3—Cd—S4—C6	138.13 (7)	C6—N2—C8—C10	106.5 (2)
N4—Cd—S4—C6	77.20 (7)	C7—N2—C8—C10	−69.5 (2)
S1—Cd—S4—C6	−72.77 (6)	C6—N2—C8—C9	−128.48 (19)
S3—Cd—S4—C6	1.71 (6)	C7—N2—C8—C9	55.5 (2)
S2—Cd—S4—C6	−133.59 (6)	C15—N3—C11—C12	0.9 (3)
N4—Cd—N3—C11	175.46 (15)	Cd—N3—C11—C12	−175.40 (15)
S1—Cd—N3—C11	−113.83 (13)	N3—C11—C12—C13	−0.7 (3)
S4—Cd—N3—C11	45.48 (14)	C11—C12—C13—C14	0.0 (3)
S3—Cd—N3—C11	104.86 (14)	C12—C13—C14—C15	0.4 (3)
S2—Cd—N3—C11	−51.79 (14)	C11—N3—C15—C14	−0.4 (3)
N4—Cd—N3—C15	−0.80 (12)	Cd—N3—C15—C14	175.94 (13)
S1—Cd—N3—C15	69.92 (13)	C11—N3—C15—C16	−179.77 (16)
S4—Cd—N3—C15	−130.78 (12)	Cd—N3—C15—C16	−3.45 (19)
S3—Cd—N3—C15	−71.39 (13)	C13—C14—C15—N3	−0.3 (3)
S2—Cd—N3—C15	131.96 (12)	C13—C14—C15—C16	179.07 (17)
N3—Cd—N4—C16	5.64 (12)	C17—N4—C16—C20	−0.5 (3)
S1—Cd—N4—C16	−120.06 (12)	Cd—N4—C16—C20	169.56 (13)
S4—Cd—N4—C16	77.17 (13)	C17—N4—C16—C15	−179.46 (15)
S3—Cd—N4—C16	141.13 (12)	Cd—N4—C16—C15	−9.43 (19)
S2—Cd—N4—C16	−62.79 (14)	N3—C15—C16—N4	8.4 (2)
N3—Cd—N4—C17	175.54 (15)	C14—C15—C16—N4	−171.00 (16)
S1—Cd—N4—C17	49.84 (13)	N3—C15—C16—C20	−170.60 (16)
S4—Cd—N4—C17	−112.93 (13)	C14—C15—C16—C20	10.0 (3)
S3—Cd—N4—C17	−48.97 (14)	C16—N4—C17—C18	−0.1 (3)
S2—Cd—N4—C17	107.11 (13)	Cd—N4—C17—C18	−170.09 (14)
C2—N1—C1—S2	7.9 (2)	N4—C17—C18—C19	0.6 (3)
C3—N1—C1—S2	−179.35 (13)	C17—C18—C19—C20	−0.5 (3)
C2—N1—C1—S1	−171.46 (15)	C18—C19—C20—C16	−0.1 (3)
C3—N1—C1—S1	1.3 (2)	N4—C16—C20—C19	0.5 (3)
Cd—S2—C1—N1	179.38 (14)	C15—C16—C20—C19	179.47 (18)

### Hydrogen-bond geometry (Å, °)

**Table:**

*D*—H···*A*	*D*—H	H···*A*	*D*···*A*	*D*—H···*A*
C18—H18···S2^i^	0.95	2.78	3.712 (2)	167

Symmetry codes: (i) *x*−1, *y*, *z*.
